# Effect of Cultural Practices on *Neopamera bilobata* in Relation to Fruit Injury and Marketable Yields in Organic Strawberries

**DOI:** 10.3390/insects11120843

**Published:** 2020-11-28

**Authors:** Hannah R. Talton, Elena M. Rhodes, Carlene A. Chase, Marilyn E. Swisher, Justin M. Renkema, Oscar E. Liburd

**Affiliations:** 1 Entomology and Nematology Department, University of Florida, Gainesville, FL 32611, USA; htalton@ufl.edu (H.R.T.); erhodes@ufl.edu (E.M.R.); 2 Horticultural Sciences Department, University of Florida, P.O. Box 110690, Gainesville, FL 32611, USA; cachase@ufl.edu; 3 Family, Youth and Community Sciences Department, University of Florida, 3026 McCarty Hall D, Gainesville, FL 32611, USA; mesw@ufl.edu; 4 London Research and Development Centre–Vineland Campus, Agriculture and Agri-Food Canada, Vineland Station, ON L0R 2E0, Canada; justin.renkema@canada.ca

**Keywords:** strawberry seed bugs, runners, strawberry cultivar, cover crops

## Abstract

**Simple Summary:**

The strawberry seed bug is a native insect that has recently been causing injury to strawberries in Florida. This study examined the effects of two common cultural practices on the strawberry seed bug: cover cropping and runner removal. Cover cropping is used only in organic strawberry production while runner removal is common to both production systems. The effects of cultivar were also examined. Lastly, small bags were tied around strawberry fruits and different numbers of nymphal and adult seed bugs were released into the bags to examine the effects of their feeding on strawberry fruits. The use of cover crops did not impact seed bug populations. The results for runner removal were inconsistent but it was clear that removing runners does not increase seed bug populations or their injury to fruit. The cultivar “Florida Brilliance” received less injury likely because its seeds are recessed in the flesh of the fruit, making them more difficult for seed bugs to access. Both adults and nymphs caused injury to ripe fruit. Adults did not feed on unripe fruit, so injury to unripe fruit is likely caused by nymphal feeding.

**Abstract:**

The strawberry seed bug, *Neopamera bilobata* (Say), is an emerging pest of organic and conventional strawberries in Florida. There is limited information on this Rhyparochromidae species. Thus, the type of injury caused is not clearly documented and management recommendations are lacking. In this study, we evaluated the effect of strawberry cultivars, cover crops, and the presence of runners on *N. bilobata* populations and yield. We also investigated the effect of select cultivars and the presence of runners on *N. bilobata* injury levels. In addition, we used fruit bagging experiments to investigate the effects of *N. bilobata* population and life stage (nymph vs. adult) on strawberry fruits. There was no effect of cover crop or cultivar on *N. bilobata* populations. In the 2017–2018 season, strawberry plots with runners contained higher *N. bilobata* populations compared with plots without runners, and adult infestation was significantly higher than nymphal infestation. In the 2018–2019 season, the trend was reversed with higher numbers of *N. bilobata* collected in plots with runners removed. In the 2019–2020 season, there was no significant difference in *N. bilobata* populations in plots with and without runners. In both 2018–2019 and 2019–2020, nymphal infestation was higher than adult infestation. Less injury was recorded in “Florida Brilliance” compared with the other cultivars tested. In the 2019–2020 season, less injury was recorded from plots without runners while the difference was not significant in 2017–2018 or 2018–2019. Releasing five and ten adult *N. bilobata* on ripe (red) fruit produced a similar level of injury while no injury to unripe (green) fruit was observed. Both adults and nymphs cause injury to ripe fruit. These findings can help contribute to the development of an integrated pest management program for strawberry *N. bilobata*.

## 1. Introduction

*Neopamera bilobata* Say (Hemiptera: Lygaeoidea: Rhyparochromidae) is native to North America but is also found in South and Central America [1]. It has been recorded from multiple plants, including pinecones, *Pinus palustris* Mill., [2], torpedograss, *Panicum repens* L., [2,3], Florida rosemary, *Ceratiola ericoides* Michx, [4], and figs, *Ficus carica* L., [5]. In strawberry (*Fragaria* × *ananassa* Duch.) fields, *N. bilobata* shelters under leaves and plastic mulch but disperses quickly when the canopy is disturbed [4]. *Neopamera bilobata* completes its life cycle on green and ripe strawberry fruit but not on strawberry leaves or flowers [6]. Populations can reach high levels in strawberry fields, as females oviposit about 300 eggs in their lifetime, and development from nymph to adulthood takes an average of 32.8 days on ripe fruit and 36.7 days on green fruit at 23 °C and 70% RH [6].

Research findings from southern Brazil [7] indicated that apical growth of the receptacle was affected by *N. bilobata* feeding at early fruit stages. Early studies conducted in Florida by Brooks et al. (1929) report that *N. bilobata* feeding causes drying and brown staining of strawberries in the early fruit stages [8]. In general, Ryparochromidae are mature seed feeders that inject enzymes into the nitrogen-rich portion of the seed so that it becomes liquefied and digestible [9]. Rani et al. (1995) found that the mandibular stylet of the seed predator *Odontopus nigricornis* (Stall) has barbs, which could help in penetrating hard seed coats, causing circular holes on the seed. Insects belonging to this order frequently cause injury and economic damage to many crops [10]. Currently, there is no economic threshold (ET) or economic injury level (EIL) established for the rhyparochromid seed feeder *N. bilobata*. The closest related seed predators in strawberries with developed thresholds are tarnished plant bug, *Lygus lineolaris* (Palisot de Beauvois) and *Lygus hesperus* (Knight), with an economic threshold of one bug per 20 plants [11].

Cultural practices may be important factors that influence the susceptibility of crops to pests, weeds, and diseases [12]. Cover crops add soil fertility, enhance crop performance, increase biodiversity, and may suppress insect pests [13,14]. In organic Florida strawberry production, summer cover crops are used primarily for nutrient management and weed and plant-pathogenic nematode management (C. Chase, personal observation) but may also affect pests during the following production season. The practice of runner-removal in annual Florida strawberry is common, as runners lead to decreased yields [15]. Daughter-plants from runners are also likely to provide shelter for pests, like *N. biolobata*, so removing runners has the potential added benefit of disrupting and potentially suppressing *N. biolobata* populations. This paper is the first to examine the effects of cover crops and runner removal on *N. bilobata*.

Plant cultivars can vary in their susceptibility to insect pests [16,17,18]. In strawberries, variations in leaf characteristics can affect the twospotted spider mite, *Tetraynchus urticae* Koch, a major strawberry pest [19]. Fruit produced by different strawberry cultivars can vary in size, sugar content, firmness, and other characteristics [20]. Hata et al. (2020) found higher numbers of *N. bilobata* on ‘Monterey’ strawberries compared with “Albion” and “San Andres” strawberries [21].

The objectives of this study were to (1) determine if cover crop choice has an effect on *N. bilobata* populations, (2) examine the effects of cultivar and runner removal on *N. bilobata* populations and fruit injury and (3) to determine the effects of adult and nymphal feeding on strawberry fruit. We hypothesized that there would be differences in *N. bilobata* populations and feeding injury among strawberry cultivars, that cover crops would not affect *N. bilobata* populations, that there would be fewer *N. bilobata* and less *N. bilobata* feeding injury in plots where runners were removed, that both adults and nymphs would injure green and ripe strawberry fruit, and that higher numbers of adult seed bugs would cause more injury to ripe strawberry fruits. New information on *N. bilobata* feeding injury and responses to cultural practices is critical for developing a management program for this important pest.

## 2. Materials and Methods

### 2.1. Cover Crops and Strawberry Cultivar

A study on the effects of cover crops and strawberry cultivars on *N. bilobata* was conducted during the 2016–2019 growing seasons in a certified organic section of the University of Florida, Plant Science Research and Education Unit (PSREU) in Citra, FL. Each season, a split plot design was used, with cover crops (Table 1) as the main plot treatments arranged in a randomized complete block with four replications. The main plots were 9.1 m × 10.7 m rectangles spaced 1.5 m apart in each block with 7.6 m alleys (buffer zones) between blocks. The cover crops were planted on 20 July 2016, 12 July 2017, and 11 July 2018 and were terminated using a flail mower on 30 September 2016, 7 September 2017, and 11 September 2018.

Strawberry cultivars (Table 2) were the sub plot treatments and were randomized within the main plots after cover crops were terminated. Strawberry plugs (Production Lareault, Inc., QC, Canada) were set in plots on 13 October 2016, 10 October 2017, and on 1 October 2018, at 0.3 m row spacing in raised double row beds covered by black plastic mulch. Each year, Nature Safe^®^ (Darling Ingredients Inc. Irving, TX, USA) 10-2-8 all season fertilizer was applied at the rate of 1681 kg/ha after bed formation and before transplanting strawberries. Weekly fertigation with a 50/50 mix of fish fertilizer (5-1-1; N-P-K) and sodium nitrate (3-0-6; N-P-K) was applied at a rate of 0.58 kg/ha.

Diseases were managed with organically approved products detailed in Table 3. Briefly, in 2017–2018 and 2018–2019, transplant plugs were dipped into a biological fungicide, RootShield^®^ Plus, before planting. In all seasons, two days before predicted rain events, RootShield^®^ Plus was applied through the drip irrigation in combination with a foliar application of an organic approved fungicide. In 2018–2019, Cueva^®^ was applied as a foliar application on 27 November and 4 December 2018 to manage an angular leaf spot outbreak. A preventative release (25 predatory mites per m^2^) of predatory mites *Neoseiulus californicus* McGregor (Koppert Biological, Howell, MI, USA) was done in mid-November during each growing season to manage twospotted spider mite, *Tetranychus urticae* Koch, population growth. When the *T. urticae* population exceeded a threshold of 10 mites per leaflet, *N. californicus* was released at the rate of 1 per 10 *T. urticae*.

#### *Neopamera bilobata* Sampling

In 2016–2017, *N. bilobata* adults and nymphs were counted in situ in a 15 cm radius around 12 randomly chosen strawberry plants per treatment. In 2017–2018 and 2018–2019, 4 plants were sampled using a 30.5 cm × 30.5 cm beat sheet and in a 15 cm radius around each plant. *Neopamera bilobata* on the plastic mulch under the plants were counted and then the beat cloth was placed next to one side of the strawberry plant and the plant was shaken for 10 s over the beat sheet and *N. biolobata* that fell on the sheet were counted. The two numbers were added together for each sample for a total per plant. In 2016–2017 and 2017–2018, *N. bilobata* were sampled from all 64 subplots. In 2018–19, *N. bilobata* were sampled from a subset of the subplots (24 out of 64) to assess cultivar effects only because data from the previous two years had indicated that cover crops had no effects on *N. bilobata* populations.

### 2.2. Strawberry Runner Removal and Cultivar Experiment

The effects of strawberry cultivar and runner removal on *N. bilobata* were assessed in field plot experiments in the organic section at the PSREU in Citra, FL during the 2017–2020 growing seasons in a separate plot from the cultivar and cover crops experiment. The plots were arranged in a split-plot design with three replications. The main plots were no runner removal and runner removal. Randomization of the main plots was restricted to prevent the movement of seed bugs between runner and no runner treatment plots. Main plots were 9.1 m × 7.5 m rectangles with a 20 m buffer zone between plots with and without runners. The main plots without runners were trimmed back every two weeks whereas those with runners were not trimmed for the entire experiment.

The subplots were the cultivars, which were randomized within each main plot. Individual subplot size was 9.1 m × 1.5 m with a total of 60 plants in each subplot. In 2019–2020, the plot length was reduced to 6.1 m × 1.5 m with 40 plants in each subplot. Three cultivars were planted each season. Winterstar™, Sensation™ “FL-127”, and “Strawberry Festival” were planted in 2017–2018. “Florida Brilliance”, replaced Winterstar™ during the 2018–2019 and 2019–2020 seasons because Winterstar™ was no longer available. Strawberry plugs were planted on 15 October 2017, 8 October 2018, and 10 October 2019 at 0.3 m row spacing in raised double row beds covered with black plastic mulch. Plants were watered three times a day at 30-min intervals using 2 drip lines for irrigation.

Fertilizer was applied on a per hectare basis through fertigation once a week using a 3-0-6 organic liquid fertilizer (Howard Fertilizer Company, Orlando, FL, USA) at the rate of 69.9 kg of nitrogen per hectare and 139.7 kg of potassium per hectare. Weeds were removed by hand as needed. Serenade^®^ Optimum and Double Nickel^®^ were used when needed to manage diseases through the season (Table 1). No insecticides were applied during the experiment. A preventative release (25 predatory mites per m^2^) of predatory mites *N. californicus* was done in mid-November during each growing season to manage *T. urticae* population growth.

#### 2.2.1. *N. bilobata* Sampling

*Neopamera bilobata* were assessed every other week, 20 November through 1 March 2017–2018 and 2018–2019 and 21 November 2019 through 12 March 2020, by vacuuming 15 randomly selected strawberry plants per plot for 10 sec each with a lithium ion handheld vacuum (Stanley Black & Decker, New Britain, CT, USA). Samples were transferred to plastic containers with water (300 mL) and detergent (1%) by removing the side panel of the vacuum, quickly pulling out the filter, and then dumping *N. bilobata* and other debris into the sample containers. Samples were then taken back to the laboratory where *N. bilobata* adults and nymphs were sorted and counted.

#### 2.2.2. Yield and Injury Evaluation

Ripe strawberries were harvested twice weekly from mid-November to March each season. Strawberries were counted, weighed, and graded in the field. Fruit was considered cull (unmarketable) if it was too small (<10 g), showed symptoms of disease, or had injury caused by insects, mammals, or abiotic factors. All culled strawberries were examined for missing achenes (seeds) due to *N. bilobata* feeding and rated by an injury rating system. Culled strawberries with no achenes removed were given an injury rating of 0. Culled strawberries with less than five achenes removed were given an injury rating of 1, those with more than five but less than 10 achenes removed were given an injury rating of 3, and those with more than ten achenes removed were given an injury rating of 5.

### 2.3. Fruit Bagging Experiments

#### 2.3.1. *N. bilobata* Colony

An *N. bilobata* colony was maintained in the laboratory beginning in late February of 2018. The colony was kept in (W30 × D30 × H30 cm) Woven Mesh Polypropylene BugDorm-1 rearing cages (MegaView Science Co., Ltd., Taichung, Taiwan) in an environmental chamber at 25 ± 1 °C, 75% RH and 14 h: 10 h, light: dark. DI water was placed in a souffle cup (50 mL) with a modified cotton wick lid and used as a water source for the insect colony. Twice a week, two or three ripe strawberries from the field were washed in deionized water and placed in the cage as a food source for the insects. Before discarding, each fruit used for feeding the colony, was carefully wiped with a 15.24 cm long plastic paintbrush (Colorations^®^, Gainesville, FL, USA) to keep all eggs and insects inside the cages.

#### 2.3.2. Adult Density Field Experiment

The effect of adult *N. bilobata* density on injury to unripe and ripe strawberry fruit was determined using two subplots of Sensation™ strawberries from experiment 2.2. Sensation™ was selected because a high amount of injury was noticed during yield sampling. This experiment was set up as a completely randomized design with split plot restrictions. The main plot treatments were *N. bilobata* densities of 0, 5, and 10 adults per plant. There were two sub plot treatments, 1 ripe and 1 unripe fruit per plant. A total of 12 plants were used for this experiment and the experiment was conducted twice per season for 2 seasons: 20–25 February 2018, 21–24 February 2018, 7–10 March 2019, and 12–15 March 2019. In 2017–2018, field caught *N. bilobata* were gathered from a near-by strawberry plot. Field caught *N. bilobata* adults were chosen at random. In the 2018–2019 season, a total of 180 adult *N. bilobata* (4 weeks old) from the colony were chosen at random for use in the experiment.

Uninjured full-sized green fruit and ripe red fruit still attached to the peduncle were covered with BugDorm (MegaView Science Co.) insect rearing bags (length 15 cm × width 6 cm). Once all fruit were covered, 0, 5, or 10 adult *N. bilobata* were released into each rearing bag. After five days, the rearing bags were collected, and injury was observed on the surface of each fruit (360° turn) and assessed using the 0, 1, 3, 5 grading system described previously in Section 2.2.2. Collection of rearing bags was adjusted to 3 days after the first trial in 2017 due to the berries being too ripe after 5 days.

#### 2.3.3. Adult vs. Nymph, Single Ripe Fruit Experiment

The effects of adults and nymphs on ripe fruit was determined in a subplot of Sensation™ strawberries from the runner removal experiment. The experimental design was a randomized complete block design with four replications (7–11 March 2018) and six replications (12–15 March 2019) of three treatments. The treatments were no *N. bilobata* (control), five adult *N. bilobata*, and five third-instar *N. bilobata*. Colony reared *N. bilobata* adults and third instar nymphs were chosen at random and placed in BugDorm insect rearing bags covering one red healthy (no physical damage or disease) strawberry still attached to the peduncle. After three days, the rearing bags with strawberries were collected and taken back to the laboratory. The entire surface of the strawberry fruits was examined and graded on a scale of 0, 1, 3, and 5 for injury as described in experiment 2.2.2.

### 2.4. Data Analysis

#### 2.4.1. Cover Crops and Strawberry Cultivar

For the 2016–2017 and 2017–2018 data, the number of *N. bilobata* per plant was averaged over the entire season and analyzed using a split plot analysis of variance (ANOVA) with the appropriate error term correction for the main plot factor (SAS version 9.4, SAS Institute, Inc., Cary, NC, USA) with cover crop type as the main plot factor and strawberry cultivar as the subplot factor. Means were separated using a least significant difference (LSD) test. In 2018–2019, since only strawberry cultivar was evaluated, *N. bilobata* per plant data were averaged over the entire season and analyzed using a one-way ANOVA and LSD means separation test. All means were considered significant when *p* < 0.05.

#### 2.4.2. Strawberry Runner Removal, Cultivar, Injury, and Bag Fruit Data Analysis

A repeated measures analysis using the GLIMMIX procedure with SAS Statistical Software (SAS version 9.4) was used to examine the effects of cultural practice (runners and no runners), selected cultivars, and insect life stage on *N. bilobata* per plot data. A split plot analysis using the GLIMMIX procedure was used to examine the effects of cultural practice and cultivars on mean total injury rating per plot. Data were examined for normality using the student panel option. A first order autoregressive covariance structure was used for all repeated measures analyses. Least squared means were separated using a Tukey–Kramer test [22]. All means were considered significant when *p* < 0.05.

The GLIMMIX procedure was used to compare the three densities of *N. bilobata* to determine effects of *N. bilobata* population and injury on Sensation™ strawberry (SAS version 9.4). Data were square root transformed. The GLIMMIX procedure was also used to compare adults vs. nymphs to determine which life stage caused more injury.

## 3. Results

### 3.1. The Effect of Cover Crops and Strawberry Cultivars on Seed Bug Populations

**Figure 1:**
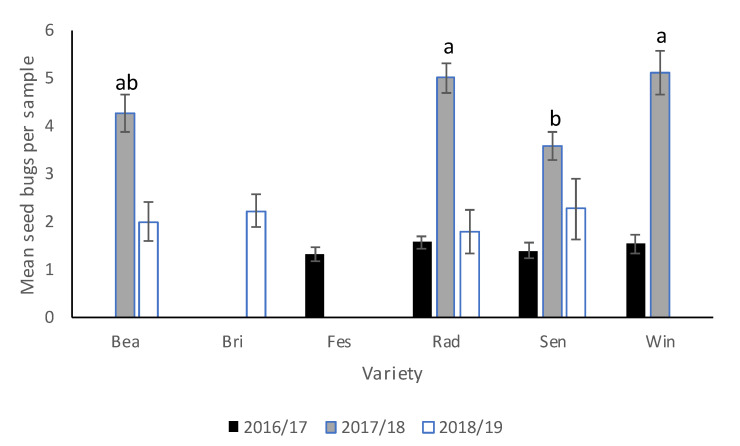
Mean (±SEM) *N. bilobata* per plant over the sample dates in each cultivar (Bea = “Florida Beauty”, Bri = “Florida Brilliance”, Fes = “Strawberry Festival”, Rad = “Florida Radiance”, Sen = Sensation™, and Win = Winterstar^TM^) for all three seasons (2016–2017, 2017–2018, and 2018–2019) in the cover crop trial conducted at the Citra Plant Science Research and Education Unit in organic strawberries. Means with different letters were significantly different at *p* < 0.05 according to ANOVA and LSD analyses.

There was no significant interaction between cover crop and cultivar in 2016–2017 or 2017–2018. In 2016–2017, there were no significant differences in *N. bilobata* numbers among cover crop (Table 4) treatments or cultivars. In 2017–2018, there were no significant differences in *N. bilobata* numbers among cover crop treatments but more *N. bilobata* were found in “Florida Radiance” and Winterstar^TM^ (Figure 1) than in Sensation™ (*F* = 4.02, df = 3, 36, *p* = 0.013). In 2018–2019, there was no difference in *N. bilobata* numbers among cultivars.

### 3.2. The Effect of Runners and Strawberry Cultivars on Seed Bug Populations, Fruit Injury and Fruit Yield

#### 3.2.1. *N. bilobata* Populations

**Figure 2:**
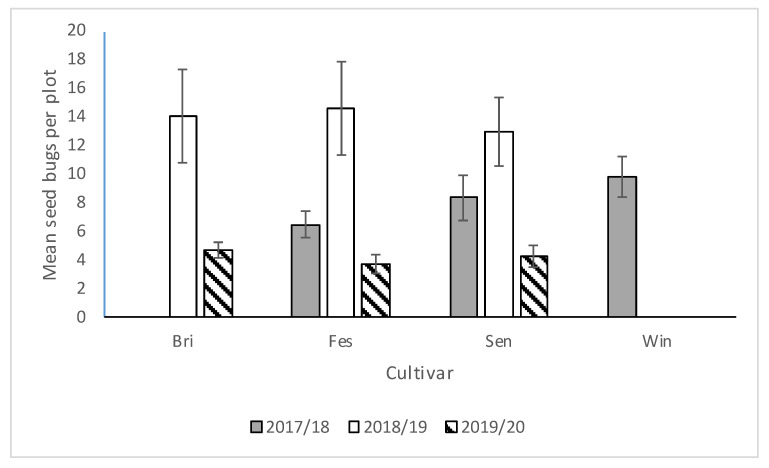
Mean (±SEM) *N. bilobata* per plot over the sample dates in each cultivar (Bri = “Florida Brilliance”, Fes = “Strawberry Festival”, Sen = Sensation™, and Win = Winterstar^TM^) for all three seasons (2017–2018, 2018–2019, and 2019–2020) in the runners vs. no-runners trial conducted at the Citra PSREU in organic strawberries. There were no differences at *p* < 0.05 according to repeated measures and Tukey’s tests.

**Figure 3:**
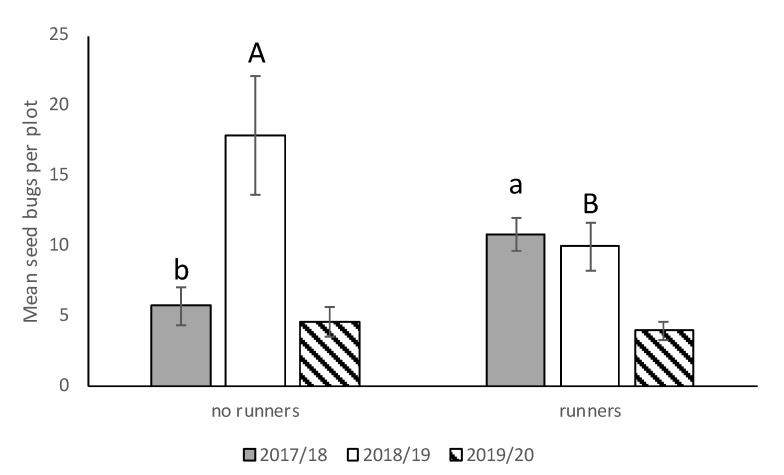
Mean (±SEM) *N. bilobata* per plot over the sample dates in plots with and without runners for all three seasons (2017–2018, 2018–2019, and 2019–2020) in the runners vs. no-runners trial conducted at the Citra PSREU in organic strawberries. Means with different lower- or upper-case letters were significantly different at *p* < 0.05 according to repeated measures and Tukey’s tests.

**Figure 4:**
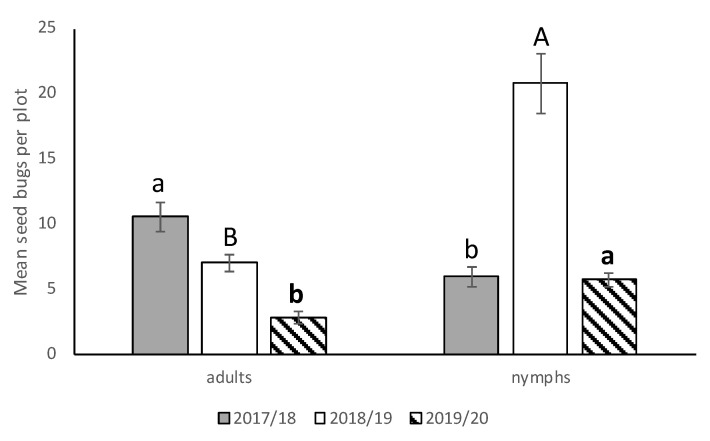
Mean (±SEM) adult and nymphal *N. bilobata* per plot over the sample dates for all three seasons (2017–2018, 2018–2019, and 2019–2020) in the runners vs. no runners trial conducted at the Citra PSREU in organic strawberries. Means with different letters were significantly different at *p* < 0.05 according to repeated measures and Tukey’s tests.

We saw no significant interactions between cultivar*runners, cultivar*stage, runners*stage, and cultivar*runners*stage for any of the three seasons, so the main effects were presented. There was no significant difference in *N. bilobata* numbers among cultivars in 2017–2018, 2018–2019, or 2019–2020 (Figure 2). In 2017–2018, there were significantly more *N. bilobata* captured in strawberry field plots where runners were not removed compared to plots where runners were removed (*F* = 16.49; df = 1,50.8; *p* = 0.0002) (Figure 3). There were approximately 1.7 times more adults captured than nymphs (*F* = 13.10; df = 1,50.8; *p* = 0.0007) (Figure 4). In 2018–2019, there were significantly more *N. bilobata* captured in strawberry field plots where runners were removed compared with plots where runners were not removed (*F* = 5.73; df = 1,24.8; *p* = 0.024). There were approximately 3.3 times more nymphs collected than adults (*F* = 15.81 df = 1,24.8; *p* = 0.0005). In 2019–2020, there were no differences in *N. bilobata* numbers among plots with and without runners. There were approximately twice as many nymphs as adults (*F* = 18.04; df = 1,53.78; *p* < 0.0001).

#### 3.2.2. Fruit Injury

**Figure 5:**
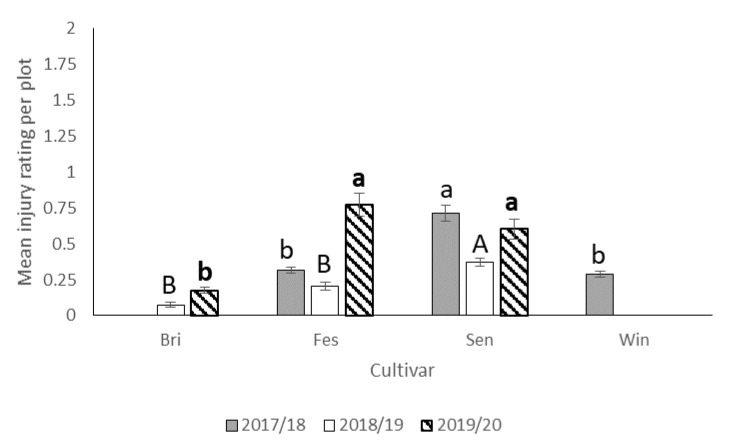
Mean injury rating (±SEM) in each cultivar for all three seasons (2017–2018, 2018–2019, and 2019–2020) in the runners vs. no runners trial conducted at the Citra PSREU in organic strawberries. Means with different letters were significantly different at *p* < 0.05 according to repeated measures and Tukey’s tests.

**Figure 6:**
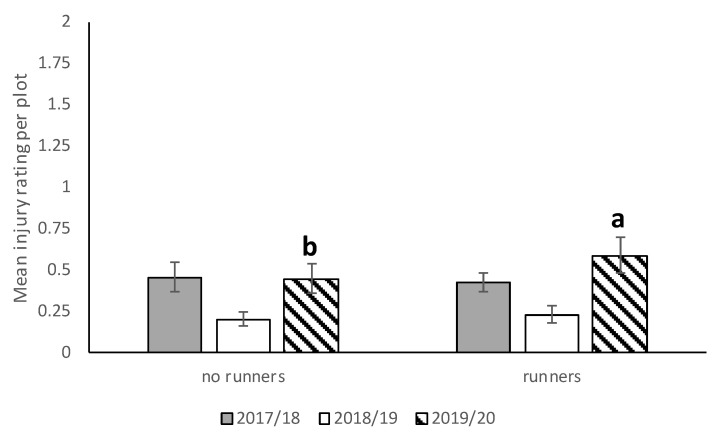
Mean injury rating (±SEM) in plots with and without runners for all three seasons (2017–2018, 2018–2019, and 2019–2020) in the runners vs. no runners trial conducted at the Citra PSREU in organic strawberries. Means with different letters were significantly different at *p* < 0.05 according to repeated measures and Tukey’s tests.

**Figure 7:**
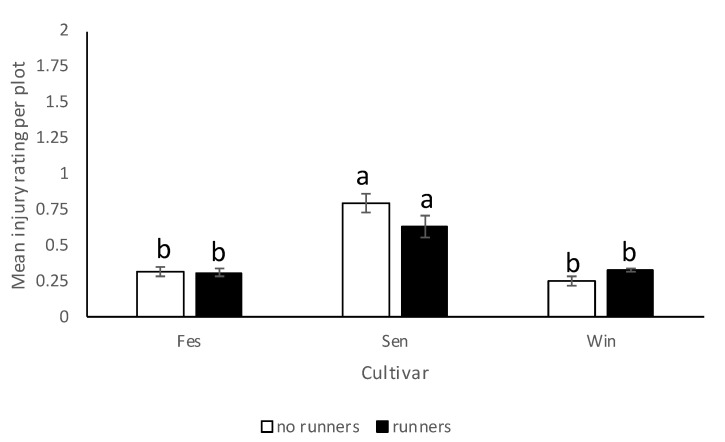
Mean injury rating (±SEM) in each cultivar in plots with and without runners for the 2017–2018 season in the runners vs. no runners trial conducted at the Citra PSREU in organic strawberries. Means with different letters were significantly different at *p* < 0.05 according to repeated measures and Tukey’s tests.

In 2017–2018, *N. bilobata* caused more injury to Sensation™ compared to “Strawberry Festival” and Winterstar^TM^ strawberries (*F* = 67.49: df = 2,10; *p* < 0.0001) (Figure 5). No difference in injury occurred in strawberry plots with runners compared to plots with runners removed (*F* = 0.93; df = 1,10; *p* = 0.36) (Figure 6). The interaction of cultivar*runners was also significant (*F* = 4.65; df = 2,10; *p* = 0.04). Higher injury was seen in Sensation^TM^ plots with and without runners compared with all the other treatments (Figure 7).

During the 2018–2019 season, there was no significant difference between strawberry plots with runners removed and those with runners (Figure 6) still attached with respect to average injury level. The highest *N. bilobata* injury was observed on Sensation™ fruit (Figure 5) and the lowest on “Florida Brilliance” fruit (*F* = 29.14; df = 2,12; *p* < 0.0001). There was no significant interaction for cultivar*runners during the 2018–19 growing season.

During the 2019–2020 season, significantly higher injury was recorded in plots with runners compared (Figure 6) to those without runners (*F* = 6.53; df = 1,10; *p* = 0.03). Higher injury was recorded on “Strawberry Festival” and Sensation™ (Figure 5) compared with “Florida Brilliance” (*F* = 40.68; df = 2,10; *p* < 0.0001). There was no significant interaction for cultivar*runners during the 2019–2020 growing season.

#### 3.2.3. Yield

**Figure 8:**
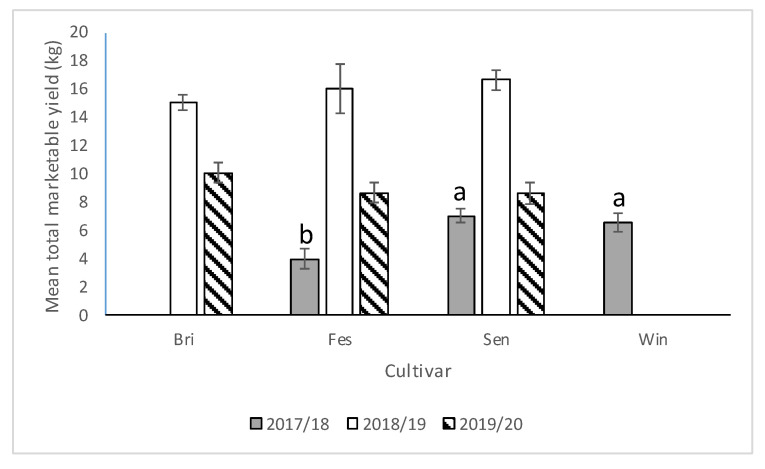
Mean total marketable yield (±SEM) per cultivar (Bri = “Florida Brilliance”, Fes = “Strawberry Festival”, Sen = Sensation™, and Win = Winterstar^TM^) for all three seasons (2017–2018, 2018–2019, and 2019–2020) in the runners vs. no runners trial conducted at the Citra PSREU in organic strawberries. Means with different letters were significantly different at *p* < 0.05 according to repeated measures and Tukey’s tests.

**Figure 9:**
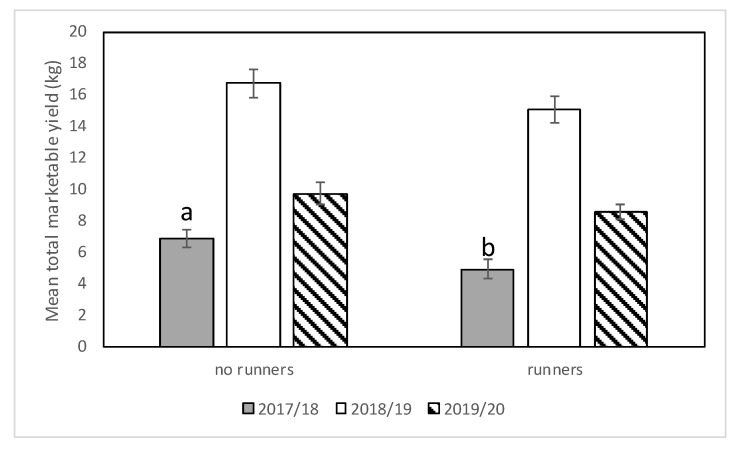
Mean total marketable yield (±SEM) in plots with and without runners for all three seasons (2017–2018, 2018–2019, and 2019–2020) in the runners vs. no runners trial conducted at the Citra PSREU in organic strawberries. Means with different letters were significantly different at *p* < 0.05 according to repeated measures and Tukey’s tests.

In the 2017–2018 season, significant differences were observed among cultivars with respect to marketable yield (*F* = 13.06; df = 2,12; *p* = 0.0010). Significantly lower yield was recorded from “Strawberry Festival” compared with Sensation™ and Winterstar™ (Figure 8). Significantly more marketable yield was recorded from strawberry plots with runners removed compared with strawberry plots with runners still attached (Figure 9). Plots that had no runners produced approximately 1.4 times as much marketable fruit yield as those without (*F* = 14.01; df = 1,12; *p* = 0.0028). No significant interaction was observed for cultivar*runners (*F* = 0.71; df = 2,12; *p* = 0.51) in 2017–2018.

**Figure 10:**
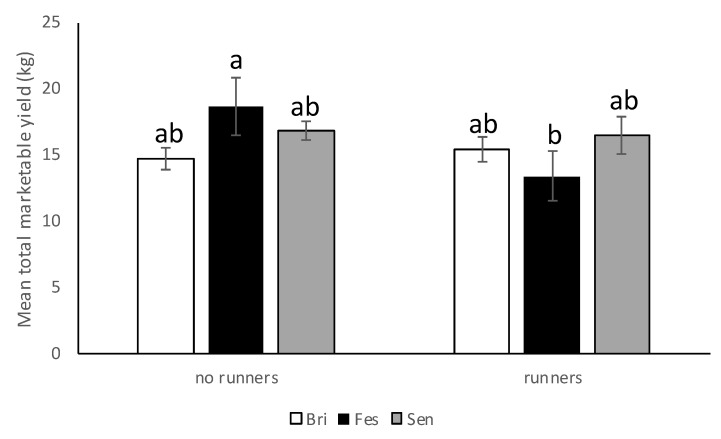
Mean total marketable yield (±SEM) per cultivar (Bri = “Florida Brilliance”, Fes = “Strawberry Festival”, Sen = Sensation™, and Win = Winterstar^TM^) in plots with and without runners for the 2018–19 season in the runners vs. no-runners trial conducted at the Citra PSREU in organic strawberries. Means with different letters were significantly different at *p* < 0.05 according to repeated measures and Tukey’s tests.

In the 2018–2019 season, there was a significant interaction between cultivar and cultural practice (*F* = 5.34; df = 2,10; *p* = 0.0265) but no differences between plots with and without runners (*F* = 4.05; df = 1,10; *p* = 0.07) nor among cultivars (*F* = 1.32; df = 2,10; *p* = 0.31). Average marketable strawberry yield (kg/per plot) was significantly higher in “Strawberry Festival” plots where runners were removed compared with “Strawberry Festival” plots where runners were still attached (Figure 10). However, no significant difference in average marketable weight (kg/per plot) was observed for “Florida Brilliance” or Sensation™ with or without runners.

In the 2019–2020 season, as in the 2017–2018 season, no significant interaction between cultivar and presence of runners was observed (*F* = 0.83; df = 2,12; *p* = 0.46). There were no differences in marketable yield among cultivars (*F* = 1.2; df = 2,12; *p* = 0.33) nor between plots with and without runners (*F* = 1.9; df = 1,12; *p* = 0.19) (Figure 8 and Figure 9).

### 3.3. Fruit Bagging Experiments

#### 3.3.1. Adult Density Experiment

There were no significant interactions between season and density, and thus data were averaged across both seasons. During the 2017–2018 and 2018–2019 seasons (Table 5), injury was significantly higher on red fruit compared with green fruit (*F =* 22.45; df = 2,22.5; *p* < 0.0001). With red fruit, injury incidence was higher with *N. bilobata* densities of five and ten when compared with the control. The control without *N. bilobata* did not have any injury. Zero green fruit were affected.

#### 3.3.2. Effects of Adults and Nymphs on Mature Fruit

In the 2017–2018 season (Table 6), significantly more injury was caused by adult *N. bilobata* compared with nymphs and by both adults and nymphs compared with the control treatment (*F =* 31.42; df = 2,33.7; *p* < 0.0001). In the 2018–2019 season, there was no significant difference in injury between adults and nymphs, but a significant difference for both treatments compared with the control (mean injury rating 0 ± 0) (*F =* 12.40; df = 2,63.1; *p* < 0.0001).

## 4. Discussion

This study examined the effects of cultivar, cover crops, and runner removal on *N. bilobata* populations. The effects of cultivar, runner removal, developmental stage, and numbers of *N. bilobata* on peudofruit injury were also examined. As hypothesized, there was no effect of the cover crop on *N. bilobata* populations, there were differences in fruit injury among cultivars, and both adult and nymphal *N. bilobata* caused injury to ripe fruit. Contrary to our hypothesis, there was no effect of cultivar on *N. bilobata* populations and adult *N. bilobata* did not injure green fruit. The effect of runner removal on *N. bilobata* populations varied among the three seasons. Fruit injury was either no different or lower in plots where runners were removed depending on the season.

Strawberry cultivar had no effect on *N. bilobata* populations with one exception, the presence of fewer *N. bilobata* on Sensation™ plants in 2017–2018 in the cover crop trial. Though statistically significant, the difference was only 1 *N. bilobata* per plot, which is likely not ecologically relevant. Another factor could have been the sampling methods used. The vacuum collects *N. bilobata* from the entire plant and the surrounding mulch and in-situ counts examined the same things. A more specific sampling method might have revealed differences. For example, [21] found differences in *N. bilobata* populations on different strawberry cultivars when they counted *N. bilobata* in-situ specifically on fruit.

In terms of *N. bilobata* injury to fruit, however, there were cultivar differences. “Florida Brilliance” had less injury than the other cultivars. This could be due to the orientation of the achenes on the fruit. The achenes of “Florida Brilliance” are positioned below the fruit surface [23], which may make them less accessible to the seed bug. In contrast, “Strawberry Festival”, Sensation™, and Winterstar^TM^ have similarly positioned achenes on the surface of the fruit that are easier for *N. bilobata* to access. The abundance and feeding study conducted by Rhainds et al. (2003) found that injury by *L. lineolaris* in strawberry fields varied greatly by cultivar [24].

There was no effect of the cover crop treatment on *N. bilobata* populations. Cover crops are terminated and then incorporated into the soil before the main crop, strawberry in this case, is planted. Therefore, the use of cover crops primarily affects soil dwelling pests like plant parasitic nematodes. Adult *N. bilobata* were not observed in strawberry fields until at least December when fruit was present in the field. It is likely that they moved from other hosts into strawberries once the fruit became available, which is perhaps the reason why cover crops had no effect on their populations.

Our findings on the effect of runners on seed bug populations varied by season. During 2017–2018, *N. bilobata* populations were higher in field plots where runners remained attached to the strawberry plant while in 2018–2019, *N. bilobata* populations were higher in plots where runners were removed. There were no differences in *N. bilobata* populations in 2019–2020. *Neopamera bilobata* injury was also higher in plots with runners compared to plots where runners were removed only in 2019–2020. Oghiakhe et al. (1991) found that vegetative growth such as canopy played a role in the infestation of cowpea, *Vigna unguiculata (L.)* Walp, by *Maruca testulalis* Geyer [25]. Similarly, the increased canopy cover and shelter provided by the presence of daughter plants from runners may result in protection for *N. bilobata* from predators and the environment (e.g., freezing, precipitation) [26]. Connecting corridors or habitat patches with interconnections, like runners could also be important for *N. bilobata* nymphal stages because they are wingless and have short dispersal distances [27]. Several factors could have contributed to the variation in results among seasons. Changes in production or pest management practices in a crop, such as replacing Winterstar^TM^ with “Florida Brilliance”, can profoundly alter the pest’s population dynamics causing shifts in pest populations [28,29]. Another change that could have affected seed bug populations was that the strawberry field was moved ~100 m) to the east between the 2017–2018 and 2018–2019 seasons. This change in location could have changed the dispersal pattern of *N. bilobata* into the field. Pasek (1998) found that patterns of insect population can be affected by local fields, wind speed, insect behavior, and vegetative composition [30].

Higher numbers of adults compared with nymphs were captured in 2017–2018 field-season. Higher adult captures could have been due to the ability of adults to withstand more adverse weather conditions due to their highly sclerotized and pruinose body surface [31,32,33], and/or lack of predators at this life stage. Adults may also have flourished because field conditions in 2017–18 were ideal with relative humidity above 65%, temperature above 25 °C, and an abundant food source [6,34,35]. Our findings are consistent with the assertion in a review by Kennedy and Storer (2000) [36] that habitat suitability for insects is dramatically affected by different factors, such as predators [37] and weather [38].

In contrast, higher numbers of nymphs were captured in 2018–2019 and 2019–2020. Research with the hemipteran insect *Lygaeus equestris* indicates that parental and offspring populations vary between habitat each year [39]. When conditions are harsh (e.g., weather, host quality), female arthropods tend to compensate by laying more eggs [40]. This was shown in work done by Norhisham et al. (2014) with the milkweed bug *Oncopeltus fasciatus* Dallas, where females must locate an adequate supply of high quality seeds before reproduction can occur because nymphal growth rates depend on the presence of seeds and their nutritional quality [40]. Since *N. bilobata* feed on strawberry achenes, it is possible that fruit quality affected host preference and abundance [41,42]. In the future, more studies need to be conducted on oviposition preference and behavior of *N. bilobata*.

The results from field studies conducted in the 2017–2018 season showed that Winterstar™ and Sensation™ cultivars produced higher yields than “Strawberry Festival” and there were higher yields in plots where runners were removed. In 2018–2019, there was an interaction between cultivar and cultural practice, where “Strawberry Festival” produced higher yields when runners were removed than when they remained attached. There were no differences in yield among cultivars nor between plots with and without runners in 2019–2020. Runner removal is a common practice in Florida strawberry production because it results in higher yields [43]. Albregts and howard (1986) reported that removing runners increased total marketable yields of “Albion” strawberries in each planting year [44]. Runner removal can reduce competition within the plant for resources between runner production and flowering, consequently improving yields [26,44].

The field density studies conducted in the 2017–2018 and 2018–2019 seasons indicated no statistical difference in mean injury rating inflicted by five or ten *N. bilobata* although the mean injury rating was numerically higher for ten *N. bilobata* per fruit. On caged *Gossypium hirsutum* L. plants, [45] found that when densities of *Creontiades signatus* Distant (Hemiptera: Miridae) were increased from 0.5 to 4 bugs per plant, cotton boll injury was increased. Several other studies indicate a decrease in yield when Hemipteran density increases [46,47].

It appears that adult *N. bilobata* do not cause injury to green fruit. This could be due to the change in fruit physiology as *N. bilobata* may need achene development at a certain stage for feeding. Fait et al. (2008) identified changes in the primary and secondary metabolites for the receptacle and achenes of strawberries, where they found major fluctuations in primary compounds in the achene like glucose, aspartate, anthocyanin synthase, and tricarboxylic acid or TCA [48]. The secondary metabolite that was in high fluctuations in the achene was catechin phenylpropanoid. It is possible that these changes within the achenes could be detected by the sensory organs of the adult *N. biolbata* and, consequently, cause the adult *N. biolbata* to feed on mature (ripe) fruit. The phenology of the achene may also play a role in why more injury occurred on ripe fruit compared with the green. The authors of [4] observed adult *N. bilobata* feeding on small dry fruit seeds instead of fleshy fruit seeds in Florida rosemary. More research, including choice experiments and gut content analysis, would need to be conducted to determine seed bug feeding behavior.

Adult *N. bilobata* caused more injury to ripe strawberries than 3rd instar nymphs in 2017–2018 but there were no differences in adult and 3rd instar nymph injury during the 2018–2019 season. Results from studies conducted by [6] showed that nymphal seed bugs could complete their life cycle on green fruit. Another seed predator study found that *Edessa meditabunda* (F.) nymphs did not reach the third instar on mature soybean pod seeds or on mature sunflower seeds [49]. It is possible that nymphs may prefer feeding on green immature achenes over mature achenes, whereas adults prefer to feed on ripe achenes compared to green.

## 5. Conclusions

Our research showed that the use of summer cover crops does not appear to affect *N. bilobata* populations. The effects of removing runners were variable among seasons, but the practice is not likely to increase fruit injury caused by *N. bilobata*. Therefore, both practices can continue to be used even on farms where *N. bilobata* is economically damaging. However, on farms where *N. bilobata* populations are high, planting cultivars with recessed achenes like “Florida Brilliance” may reduce fruit injury. Both *N. bilobata* nymphs and adults cause injury to ripe strawberries in Florida and nymphs are also capable of injuring unripe strawberries. Therefore, both life stages should be targeted for management to prevent economic damage.

## Figures and Tables

**Table 1:** Cover crop treatments.

Treatment	Cover Crop(s)
Hairy indigo	*Indigofera hirsuta* L.
Sunn hemp	*Crotalaria juncea* L. Cv. Tropic Sun
4-way mix	Hairy indigo Sunn hemp cv. AU Golden American jointvetch (*Aeschynomene americana* L.) Slender leaf rattlebox (*C. Ochroleuca* G. Don.)
Weedy control	No cover crop

**Table 2:** Strawberry cultivars planted each season.

Treatment	2016–2017	2017–2018	2018–2019
1	“Strawberry Festival”	“Florida Beauty”	“Florida Beauty”
2	“Florida Radiance”	“Florida Radiance”	“Florida Radiance”
3	Sensation^TM^ “Florida127”	Sensation^TM^ “Florida127”	Sensation^TM^ “Florida127”
4	Winterstar^TM^	Winterstar^TM^	“Florida Brilliance”

**Table 3:** List of fungicide products used for disease management and the label rate used for each.

Brand Name	Active Ingrediant	Application Type	Rate	Company
Rootshield^®^ Plus WP	*Trichoderma harzianum* Rifai strain T-22 and *T. virens* strain G-41	root dip through drip irrigation	47 mL/100 L water 1.1 kg/ha	BioWorks^®^ Victor, NY
Actinovate^®^ AG	*Streptomyces lydicus* WYEC 108	foliar	0.56 kg/ha	Valent USA LLC St. Louis, MO
Double Nickle^®^ LC	*Bacillus amyloliquefaciens* strain D&$&	foliar	3.36 kg/ha	Certis USA LLC Columbia, MD
Regalia^®^	Extract of *Reynoutria sachalinensis*	foliar	2.34 L/ha	Marrone^®^ Bio Innovations Davis, CA
Serenade^®^ Optimum	QST 713 strain of *Bacillus subtillis*	foliar	1.5 L/ha	Bayer Cropscience LP Research Triangle park, NC
Cueva^®^	Copper octanoate (copper soap)	foliar	19 L/ha	Certis USA LLC Columbia, MD

**Table 4:** Mean ± SEM *N. bilobata* per plant over all sample dates in each cover crop treatment.

Cover Crop	2016–2017 Season	2017–2018 Season
Hairy indigo	1.5 ± 0.2	4.5 ± 0.3
Sunn hemp	1.3 ± 0.1	5.4 ± 0.4
4-way mix	1.6 ± 0.2	4.1 ± 0.5
Weedy control	1.3 ± 0.1	4.1 ± 0.4

**Table 5:** Mean ± SEM injury rating per fruit for the adult density experiment. Means with different letters were significantly different at *p* < 0.05 according to ANOVA and Tukey’s tests.

N. bilobata per Fruit	Green Fruit	Red Fruit
0	0 ± 0	0 ± 0 a
5	0 ± 0	2.3 ± 0.7 b
10	0 ± 0	3.4 ± 0.7 b
Total	0 ± 0 A	1.6 ± 0.4 B

**Table 6:** Mean ± SEM injury rating per fruit for the adults vs, nymphs experiment. Means with different letters were significantly different at *p* < 0.05 according to ANOVA and Tukey’s tests.

*N. bilobata* (5 per Fruit)	2017–2018 Season	2018–2019 Season
control (none)	0 ± 0 a	0 ± 0 A
3rd instar nymphs	1.3 ± 0.4 b	1.8 ± 0.4 B
adults	3.5 ± 0.5 c	1.2 ± 0.4 B
